# Cleaning Teeth

**Published:** 1905-05-15

**Authors:** N. S. Hoff


### CLEANING TEETH.

BY N. S. HOFF, D.D.S.

Read before the Central Michigan Dental Society, March 28, 1905.

No operation required of the dentist is of more value to the patient than the cleansing of the teeth; and yet of all operations it is the most neglected or most carelessly performed. In college it is the one operation that students think they need not learn to do carefully. In practice it is the one operation they systematically avoid if possible. This does not of course apply universally, but it is so generally true that it is a statement which can not be successfully challenged. It has never been clear to me why this is so. I have asked many dentists why they do not clean teeth more thoroughly, and some have said that they could not afford to do it for the fees patients are willing to pay. Others that they do not like to do that kind of work. The student in college does not want to do it because he thinks it is beneath his dignity, especially if he happens to be a senior. Because of this neglect of so important a matter the teeth and gums of our patients degenerate, and our best efforts in the line of operative and prosthetic restorations fail. There seems to be some encouraging signs that this subject is to receive more careful attention in the future. There has been during the past year or two a gradual development of a new specialty in dentistry, which results from the efforts being made to cure the bad cases of pyorrhœa alveolaris. After the long discussion as to the etiology of this disease, which is not yet clearly made out, we have come to the conclusion that it is essentially an operative disease. And all treatment at the present time by modern operators, is directed primarily at a careful cleansing of the teeth, and then to the adoption of hygienic local treatment, and almost entirely without medicaments. Several well-known dentists have given up general practice and have resorted to the treatment of diseased gums only, and seem to be succeeding very well. One of these men has not only given up his time to the treatment of diseased gums, but begins his work with the deciduous teeth as soon as they are only partially erupted and follows each patient along with regular periodic treatments through his life. The claim is that much or practically all decay of the teeth can in this way be prevented or hindered at least, and that the gums will be kept in the best possible condition.

Why should the teeth be cleaned? This is the question that patients ask and it is one that very few dentists can or will answer adequately. The most recent researches as to the causes of decay of the teeth has established the fact that bacterial consequences are the immediate cause of dental caries; and as a corrolary that bacteria can not exist or maintain a vigorous function except in the presence of a suitable medium, such for instance as may be found in the mouth, and especially amidst the accumulated carbohydrate food stuffs in and about irregular and decayed teeth. It has been found that the bacteria which do the most injury attach themselves in a kind of glutenous plaque to favorable positions on the teeth, and by a vigorous reproduction of their kind they produce a serious decomposition of the hard tooth structure by the erosive acid ferments which they produce. It then is very plain to be seen that if the teeth can be protected from the accumulation of these disease-producing agents they will be freed from decay. Hence the effort to thoroughly clean teeth and to polish and fill them so that the patient can by the ordinary mouth toilet keep them free from such menacing reagents would seem to be a logical if not an imperative demand on every dentist who professes to have the welfare of his patients at heart. This ought to be a sufficient answer and inducement to any patient who believes in preserving his body from the attacks of disease.

The question of how soon should this work be begun and how often it should be done will answer itself for each individual patient. Some patients do not need to consult their physicians more than once or twice each year, while others must see their's two or three times each year, and many retain their physicians by the year. To prevent their decay, the teeth should be kept scrupulously clean, and no decayed tooth as such should be allowed in the mouth at any time. Therefore to be consistent we must begin with the babes and keep it up as long as the teeth remain in the mouth. How often? Why just as often as occasion requires. If the cleaning is well done each time, and there is no vitiated secretion from the oral membranes to vitiate the saliva or to attach itself to the teeth, and the patient will use the brush and powder systematically, once each month may be often enough, except during those periods when the teeth are especially liable to attacks from the rapid development of disease producing agents. In many mouths, and at certain ages when the mouth is immune, once in three or six months may be often enough for the dentist to give the teeth thorough cleaning and polishing. There can be, however, no set rule about such a matter. Existing conditions or accidental conditions may call for more or less variation of any time rule which may be set. With intelligent adult patients the matter may be left largely to their discernment. There is another condition which makes a more imperative demand for this prophylactic treatment, and that is the so-called pyorrhœa alveolaris, which sets in with many people at the age of forty-five to fifty years. It may appear much younger than this in persons of sedentary habits or of rheumatic diathesis. In these cases the thorough removal of all extraneous deposits on the crowns and roots is an absolute necessity in order that the roots may be polished so that the irritated gums may heal and close up around the root and thus prevent the accumulation of pathogenic or destructive bacteria. No more ideal incubator can be imagined than can so generally be found in the pockets about the roots of teeth affected with this disease. The thorough eradication of the conditions favoring disease here is highly important, and these patients must be seen as often as necessary to keep down the irritating causes and the sooner the treatment is begun and the more thoroughly it is made, the better the chances for permanently eradicating it. I need not here refer to the necessity for prompt treatment at the first indications of disease, as every dentist knows how hopeless an undertaking it is to try to cure a case of pyorrhœa that has made great progress. If the treatmeat is not begun before the teeth become loosened and mal-occlusion takes place the chances of a cure are generally negative. These two reasons then are surely enough, so far as consequences of the greatest importance to the patient is concerned, to warrant the dentist in devoting sufficient time to every patient who comes into his hands to prepare his teeth for being kept free from all injurious contaminations.

The value of this service to the patient should amply justify any reasonable fee, that will compensate the dentist for his time and skill, just as fully as for any other operation. This, in passing, is one of the fees which can not be fixed on the basis of "so much per." And any one who attempts to do it on such a basis will either give a perfunctory service or discriminate to his own or his patient's disadvantage. Make the service all that the case requires and then adjust the fee on a proper business basis. A few words as to how this work shall be done may not be out of place, yet it would require a small volume to adequately detail all methods that may be advantageously used. In the first place a proper equipment of instruments and appliances are necessary. There is no single set of instruments perhaps that will fulfill all the requirements of every case that may come to one's care. Even if the cases were all similar there is so much difference in the method which each one employs for handling his instruments that he can not manipulate all instruments with the nice tactile sense which is very necessary here. A sufficient variety of scalers should be on hand to reach every part of any tooth, whether in proper position or not. In pyorrhea cases of long standing the teeth are often irregular and the deposits far beneath the gum and out of reach with any of the ordinary instruments. It is scarcely probable then that any one could have too many instruments. It is also highly important that these instruments shall be well polished so that they may be kept clean and also that they shall be sharp, and small enough that they will reach the ultimate deposits with the least possible laceration of the gums.

We may classify the treatment under the two heads of operative and prophylaxis. Although the therapeutic treatment is frequently indicated we have no time or space here to consider it. By operative treatment we mean, what we might truly call the disinfectant treatment, as its final purpose is to rid the teeth of all extraneous deposits of every sort and restore them to as nearly the normal condition as is practicable, so that the normal function of the tissues can have a chance to bring about a healing result, which they will always try to do. The first operation is usually the scaling of the hard concretions from the teeth. The care with which this part of the work is done will have much to do with all subsequent operations. As we have already indicated suitable instruments for this purpose should be provided, and these should be of good quality and a sufficient variety of forms to reach every possible position implicated. They should be sharp and delicate rather than large dull and clumsy instruments such as we generally find in the dentist's outfit. They should be preferably cone-socket points in wood handles to give lightness and bulk to the handle so as to accentuate the touch. The wood handles are objectionable because they can not be sterilized advantageously. If we could have aluminum handles of the same patterns as our wood handles they would be all that we could wish. The points should be taken from the handles after using and thoroughly sterilized before using on another patient. This, of course, is best done in a steam sterilizer if one is convenient, otherwise in some good chemical sterilizer. We prefer for this purpose a five percent solution of lysol in distilled water. The instruments can remain in this solution as long as may be desirable without injury from chemical corrosion, and this solution is one of the best known bactericides. They can be boiled in this solution, but the odor is objectionable in the office. A five percent carbolic solution is also a good sterilizer, but its corrosive action will soon take the delicate edge off the instruments. The other powerful sterilizers are even more objectionable from this standpoint. Before beginning to scale the teeth the mouth should be thoroughly sterilized. If the patient has brought his tooth brush with him, have him thoroughly brush the teeth with a good powder and a little soap. Then have him hold for two or three minutes a mouthful of some agreeable antiseptic lotion. In scaling the teeth it is a decided advantage to confine the work to a single tooth, if practicable. By so doing the time wasted in changing instruments is avoided, and the work is likely to be more thoroughly done. This can not always be followed, as there may be excessive hemorrhage or the tooth may be so sensitive that the patient can not endure continued irritation. Preferably, it is better to begin with the lower third molar and work forward to the cuspid and then take the opposite side in a similar manner, leaving the anterior teeth to the last. By this system, if there is much hemorrhage, it is confined to the back part of the mouth and does not obscure the teeth to be operated on last. After the lower teeth are scaled the upper can be managed in the same manner. In many cases there will be so much congestion of the gums that it may not be practicable to completely scale all the teeth at a single sitting. In such cases it is best to scale the teeth of both jaws on one side of the mouth only at the first and the opposite side at the next sitting. In this way the patient will have one side of the mouth free from the after pains to use, and in a day or two the soreness will have subsided and the opposite side can be operated on. In some exceedingly bad cases of pyorrhea it may not be possible to operate on more than two or three teeth at a sitting. Very often where the patient is easily available this method is much preferable, as the operable area being small it can be cocainized and thoroughly done and allowed to heal before another operation is made on the adjoining or opposing teeth.

In scaling the teeth the instrument used in every case should be adapted to the condition. Some operators take pride in being able to do a large number of things with a very few instruments. A post hole can be dug with a scoop shovel if there is plenty of muscle behind it, but a properly-constructed spade will accomplish the object in a superior manner and with much less effort. The question as to whether we should push the calculus off the tooth, or pull it off, has been much discussed. Both methods are necessary and a careful diagnosis of the condition will generally indicate the direction and at the same time indicate the kind of instrument best adapted to the purpose. As a general principle it is best to use the traction method, as there is less danger of mutilating the gum. Care should always be taken not to cut grooves in the enamel or cementum with the sharp angles of the scalers, as it will be almost impossible to polish these so that they will not serve as places of attachment for new deposits. And again such places are likely to be sensitive. A good knowledge of the anatomy of the root as to its root form, as well as to the alveolus and gum structure, will be very helpful in determining the extent of the deposit, and the best way to apply the force necessary to remove it.

The use of chemical reagents for softening the calcareous deposits on teeth that are difficult to scale is a hazardous proceeding and should not be resorted to except in extreme cases where it is impracticable to remove them by instrumentation. Sulphuric acid or other similar corrosive acids only are effective for this purpose. No very definite result can be expected from a weaker solution than ten percent. And an acid of this strength will produce considerable escharotic, if not caustic and destructive action on the peridental structures, which should be preserved as nearly in their normal form as is practicable. The gum tissue will stand considerable mutilation and heal to a good scar tissue, but it is always less resistant to the attacks of disease afterward, and is likely to shrink away exposing more or less of the tooth root. It is therefore desirably to puncture or bleed the gum as little as possible, except in those turgid conditions where it can't be avoided and is sometimes indicated to relieve the plethora and promote normal absorption and resuscitation of the trophic functions.

After all deposits that can be reached with the scalers have been removed it remains to remove all roughness of the roots due to the attachment of the calculus which can not be removed by means of the scalers. This is best done by using some abrasive material on wood points or engine polishing points of wood or rubber and the small engine brush wheels. The crowns of the teeth may be fairly well polished with the engine polishers, but it is impracticable to use them on the roots where there is the greatest need for careful and thorough work. Small wood points made from the ordinary orange wood sticks are very useful for this purpose. For use on the lingual surfaces they should be cut short and inserted in a suitable "hand porte polisher." The one suggested by Dr. D. D.

Smith, of Philadelphia, is a very useful and valuable instrument. With these points all the surfaces of the roots can be treated and made tolerable to the delicate sorrounding soft tissues. The approximal surfaces can be polished by means of thin cotton tape impregnated with fine pumice. or if the teeth are in close contact, one or two strands of floss silk can be passed through the teeth with the pumice until these surfaces are made smooth. A rubber band or a piece of chamois skin with the pumice will be found helpful. After the teeth are thus thoroughly cleaned they should be thoroughly irrigated or sprayed to remove all loose particles lodged in the tissues. If any treatment is required it should be made at this time. Small amounts of medicine may be applied with the wood points in the porte polisher, or dropped in the bottom of the cavity by means of a hypodermic or Dun's syringe. If the scaling and polishing has been thoroughly done very little medication or surgical interference is again permissible. The continued use of strong drugs is almost as bad practice as the continual surgical interference with the healing wound. The general surgeon does not keep opening a wound, neither does he insist on doping it with strong escharotics or poisonous disinfectants. Dentists think the gums need continual prodding to induce them to do their duty. We have had the best results from relieving the irritation and then allowing nature to take her own course, assisting only with such mild interference as will promote normal activity rather than abnormal inflammation.

We can not leave this subject without calling attention to the importance of following up such a thorough treatment by periodically going over the mouth to keep it in such condition that the patient can keep it sanitary by the ordinary mouth toilet, which is another subject of great importance, and one which has been much talked and written about, and which is not practiced as consistently by any of our patients as it should be.

